# Sequential Anti-HER2 (Human Epidermal Growth Factor Receptor 2) Therapy and Late-Line Tucatinib in Patients With HER2-Positive Breast Cancer and Brain Metastases: A Real-World Case Report

**DOI:** 10.7759/cureus.117705

**Published:** 2026-10-09

**Authors:** Purnima Thakur, Manish Gupta, Lalit C Kant, Priya Mehta, Vipulkumar Thummar

**Affiliations:** 1 Radiation and Oncology, Indira Gandhi Government Medical College & Hospital, Shimla, IND; 2 Medical Affairs, Zydus Lifesciences Limited, Ahmedabad, IND

**Keywords:** ado-trastuzumab emtansine, brain metastases, her2-positive breast cancer, sequential anti-her2 therapy, tucatinib

## Abstract

Human epidermal growth factor receptor 2 (HER2)-positive breast cancer is associated with a high incidence of brain metastases (BMs), particularly among young and heavily pretreated patients, with intracranial disease remaining a major determinant of survival and quality of life (QoL). Tucatinib, a HER2-selective tyrosine kinase inhibitor (TKI), combined with trastuzumab and capecitabine, has demonstrated significant progression-free survival (PFS) and overall survival (OS) benefit, including in patients with active BMs.

We present the case reports of two patients, both younger than 50 years, with HER2-positive metastatic breast cancer and biologically aggressive disease who progressed on prior anti-HER2 therapy (trastuzumab, lapatinib, and ado-trastuzumab emtansine (Ujvira™) in Case 1 and trastuzumab in Case 2) with worsening central nervous system involvement. Both were subsequently treated with tucatinib-based combination therapy (Tukavo™) later in the treatment sequence, once it became available and affordable, and showed clinical and radiological improvement. Case 1 had improvement in right-sided limb weakness and speech with no further seizures and remained clinically stable for seven months, and Case 2 had complete resolution of headache and vomiting, with Eastern Cooperative Oncology Group (ECOG) performance status improving from 2 to 1. QoL was assessed clinically from symptoms and performance status rather than with a validated instrument. This real-world experience illustrates the potential benefit of tucatinib-based regimens in young patients with aggressive HER2-positive breast cancer and BMs, even when used later in the treatment sequence. These findings are consistent with pivotal trial data in a resource-limited, real-world setting.

## Introduction

Breast cancer is the most commonly diagnosed cancer in women worldwide, with approximately 2.3 million new cases and 666,000 deaths in 2022 [1]. Human epidermal growth factor receptor 2 (HER2)-positive breast cancer constitutes approximately 15%-20% of all breast cancers and is characterized by biologically aggressive, amplification-driven proliferative behavior [2]. Brain metastases (BMs) develop in up to 30%-50% of patients with HER2-positive metastatic breast cancer (MBC), representing the highest central nervous system (CNS) tropism among all breast cancer subtypes, and remain a major determinant of morbidity, neurological decline, and impaired quality of life (QoL) [3].

Sequential anti-HER2 agents, including trastuzumab, lapatinib, ado-trastuzumab emtansine (T-DM1) (Ujvira™), and pertuzumab, have substantially improved outcomes in HER2-positive MBC [4-6]. However, intracranial activity is heterogeneous, and CNS progression remains common after exhaustion of multiple treatment lines, where access to newer agents is often limited [7]. Tucatinib, a reversible, highly selective HER2 tyrosine kinase inhibitor (TKI), was developed to specifically address this unmet need. In the pivotal HER2CLIMB trial, the addition of tucatinib to trastuzumab and capecitabine significantly improved progression-free survival (PFS) and overall survival (OS) in pretreated HER2-positive MBC, including in patients with active or stable BMs, with preserved QoL [8-12].

Although trial data firmly establish the efficacy of tucatinib-based therapy (Tukavo™), real-world evidence from resource-constrained settings, where access to anti-HER2 agents is frequently delayed by cost and availability, remains limited. Younger patients often present with biologically aggressive, treatment-refractory disease and early CNS dissemination; in a recent real-world cohort, patients with BMs were significantly younger at diagnosis than those with distant metastases at other sites (mean 52.4 versus 61.1 years) [13]. We describe two such patients who received tucatinib-based therapy after progressing on prior HER2-directed therapy. Patients enrolled in HER2CLIMB had received prior trastuzumab, pertuzumab, and T-DM1 and were excluded if treated with lapatinib within the preceding 12 months [8]; in contrast, neither of our patients had received pertuzumab, and one started tucatinib immediately after lapatinib. While the efficacy of this regimen is established by randomized trial data, these cases describe clinical observations in a population underrepresented in that trial.

## Case presentation

Case 1

Durable Intracranial Control With Late-Line Tucatinib-Based Therapy in a Heavily Pretreated Patient

A 47-year-old woman was diagnosed in November 2022 with locally advanced carcinoma of the right breast (cT4bN1M0). Core biopsy revealed invasive ductal carcinoma that was estrogen receptor (ER)-positive, progesterone receptor (PR)-positive, and HER2/neu 3+ by immunohistochemistry, reflecting biologically aggressive disease in a young patient [2]. Germline BRCA testing was negative, and baseline positron emission tomography-computed tomography (PET-CT) showed no evidence of distant metastases.

The patient received one cycle of neoadjuvant chemotherapy with docetaxel, carboplatin, and trastuzumab (TCH), omitting pertuzumab owing to financial constraints, achieving a clinical response of more than 70% reduction in lesion size on clinical examination. In view of this early response and patient preference, she then underwent right modified radical mastectomy in December 2022; histopathology revealed ypT2N1 disease with lymphovascular invasion, perineural invasion, and a positive lateral resection margin. She completed five further cycles of adjuvant TCH and received adjuvant radiotherapy to the right chest wall, axilla, and supraclavicular fossa, delivered after the second adjuvant cycle in a sandwich sequence, followed by maintenance trastuzumab and endocrine therapy with tamoxifen.

During her eighth cycle of maintenance trastuzumab (January 2024; 14 trastuzumab cycles in total, including six administered with TCH), the patient developed neurological deficits; magnetic resonance imaging (MRI) of the brain revealed multiple metastases involving the bilateral cerebellum and the left parietal and occipital regions. She received whole-brain radiotherapy (50 Gy in 25 fractions, completed March 2024) and underwent ovarian ablation by pelvic radiotherapy to a dose of 12 Gy in 4 fractions, to achieve permanent ovarian suppression in premenopausal hormone receptor-positive disease. Systemic therapy was switched to lapatinib plus capecitabine in March 2024 owing to non-availability of tucatinib and unaffordability of trastuzumab deruxtecan at that time (Figure 1).

**Figure 1:**
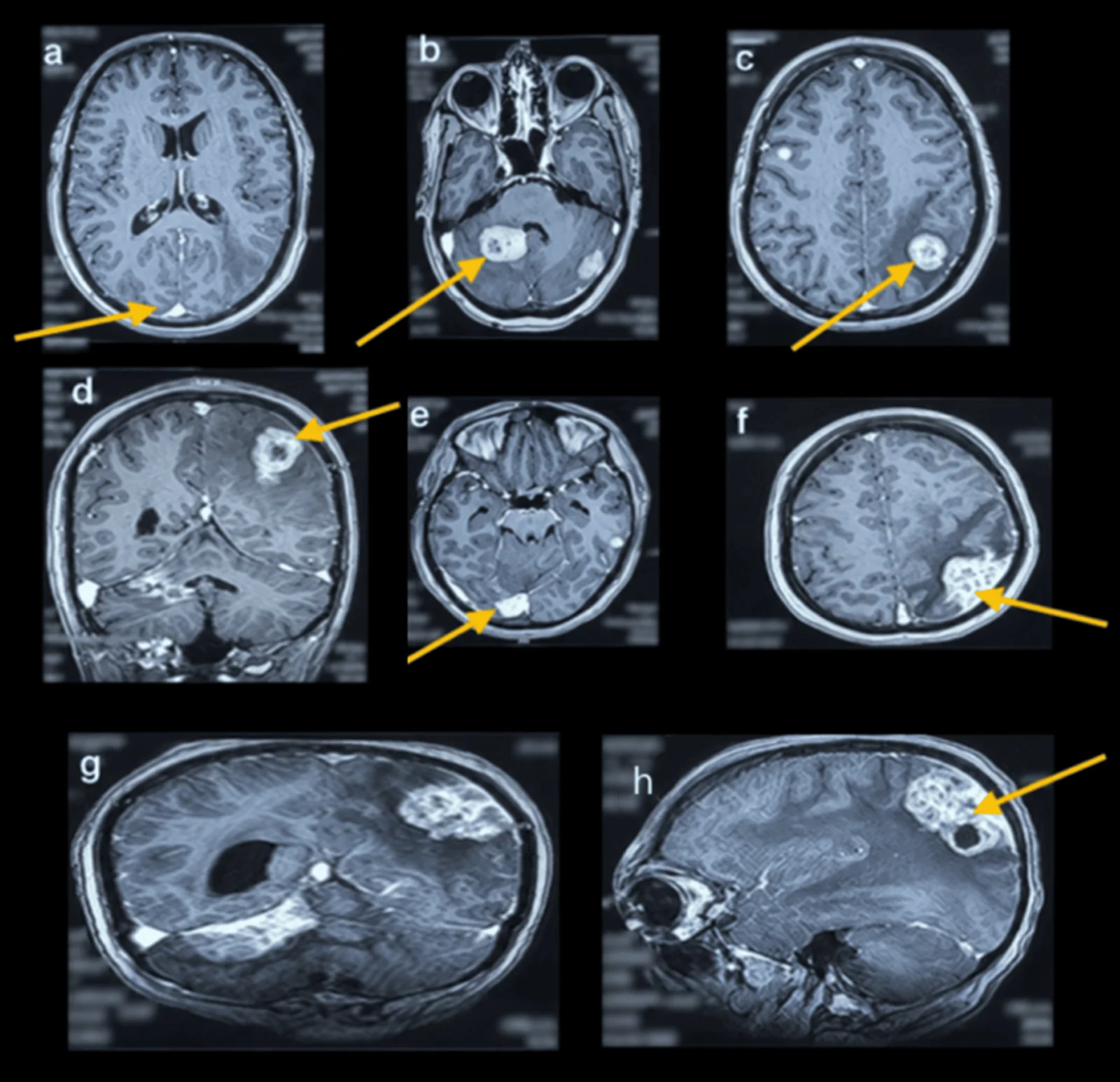
Contrast-enhanced T1-weighted MRI brain showing intracranial changes during tucatinib-based therapy in Case 1 (a, b) Baseline, before initiation of tucatinib (June 2025); (c–e) during tucatinib-based treatment (October 2025); (f–h) last follow-up on tucatinib (January 2026), showing a reduction in lesion size and perilesional edema. Straight arrows indicate the intracranial metastatic lesions. Standardized lesion measurements were not performed.

Symptomatic control persisted for approximately six months before recurrent seizures and worsening neurological symptoms (MRI showing lesions in the right cerebellum and left parietal region, the largest measuring 1.9 × 3.5 × 1.5 cm) prompted a switch to ado-trastuzumab emtansine (T-DM1; 3.5 mg/kg, September 2024), an agent with only modest reported intracranial activity [14]; the patient progressed clinically and radiologically after three months. Lapatinib and capecitabine were reintroduced in December 2024, while funds for pertuzumab were being arranged, achieving a further six months of disease stabilization. Following additional neurological deterioration (right-sided upper and lower limb weakness and slurred speech, managed conservatively), and with tucatinib becoming available, the patient was commenced on tucatinib (300 mg twice daily), trastuzumab, and capecitabine in July 2025.

The patient showed clinically evident improvement after two cycles, with recovery of right-sided limb power and speech and no further seizures, followed by continued improvement and clinical stability for seven months (July 2025 to February 2026). Treatment was well tolerated, with no grade 3 or higher adverse events and no dose reductions. She then developed recurrent neurological symptoms. MRI showed stable-sized lesions with significant perilesional edema, which were considered more suggestive of treatment-related change than true progression based on the stable lesion size and subsequent clinical improvement. However, the two could not be definitively distinguished because advanced imaging was not performed. In view of the neurological worsening, tucatinib-based therapy was discontinued, and the patient was transitioned to dual HER2 blockade with three-weekly trastuzumab and pertuzumab, with marked symptomatic improvement at the last follow-up (April 2026; total follow-up of 41 months from diagnosis).

Case 2

Rapid Neurological Recovery With Tucatinib-Based Therapy in Heavily Metastatic HER2-Positive Breast Cancer

A 49-year-old woman presented in October 2024 with a two-year history of an untreated breast lump, having defaulted on surgery advised at a local hospital, that had progressively ulcerated and begun to bleed. Examination revealed a 6 × 6 cm ulcerated right breast mass. Biopsy confirmed invasive ductal carcinoma that was ER/PR-negative, HER2/neu 3+, with a Ki-67 proliferation index of 55%, indicating highly aggressive tumor biology in a young patient. Staging confirmed cT4bN3cM1 disease with multiple pulmonary metastases and mediastinal lymphadenopathy.

The patient received hemostatic single-fraction radiotherapy to the bleeding lesion, followed by six cycles of TCH, achieving a clinical response exceeding 70% reduction in breast mass size on clinical examination. PET-CT confirmed decreased metabolic activity in the breast primary but progression of pulmonary metastases. The patient subsequently received palliative radiotherapy (30 Gy in 10 fractions) followed by a mold brachytherapy boost to the residual chest-wall lesion (21 Gy in seven fractions, twice daily; total equivalent dose in 2 Gy fractions of approximately 61 Gy) and continued maintenance trastuzumab.

The patient later developed a headache, which was dull, continuous, in the parieto-occipital region with a pain score of 4 out of 10 on the Visual Analog Scale (VAS), for which an MRI (June 2025) was done. The MRI suggested a peripherally enhancing lesion measuring 6 x 6 mm in the right occipital region with perilesional edema, suspected to be neurocysticercosis on the basis of its small size, ring enhancement, and perilesional edema in an endemic region. After eight cycles of trastuzumab, the patient developed worsening headache to a pain score of 6 out of 10 (on VAS) and vomiting, and repeat MRI (September 2025) revealed large enhancing lesions in the right parieto-occipital (5.1 × 4.5 × 5.4 cm) and the right temporal (7.9 × 6.3 × 5.6 cm) regions with significant perilesional edema, consistent with progressive BMs. This diagnosis was based on rapid interval growth, multiplicity, and imaging features in the setting of known metastatic disease, and histological confirmation was not obtained due to the patient’s refusal of the procedure. The patient received whole-brain radiotherapy (56 Gy in 28 fractions, September to November 2025) and was commenced on tucatinib, trastuzumab, and capecitabine in November 2025, immediately after completion of radiotherapy (Figure 2).

**Figure 2:**
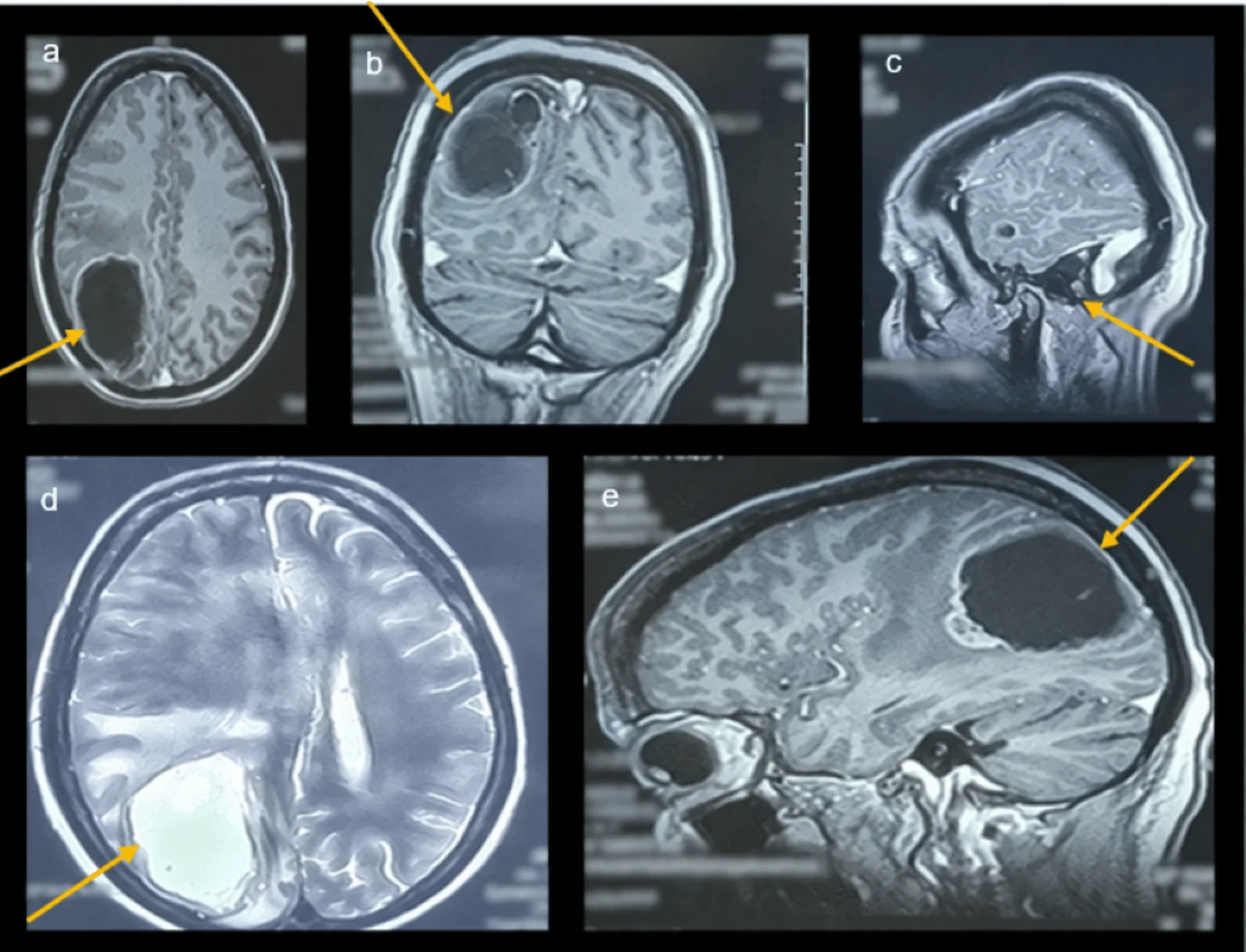
MRI brain showing changes during treatment in Case 2 (a) Baseline, before whole-brain radiotherapy (September 2025; contrast-enhanced T1-weighted); (b, c) during treatment (December 2025; contrast-enhanced T1-weighted); (d, e) follow-up after tucatinib-based therapy (March 2026; d, T2-weighted; e, contrast-enhanced T1-weighted). Straight arrows indicate the intracranial metastatic lesions and associated perilesional edema.

The patient experienced improvement in neurological symptoms, with complete resolution of headache and vomiting within the first two cycles of tucatinib. At the last follow-up (April 2026; five months on tucatinib), she remained asymptomatic, with improvement in the Eastern Cooperative Oncology Group (ECOG) performance status from 2 to 1 [15], while continuing tucatinib-based therapy. Treatment was well tolerated, with no grade 3 or higher adverse events and no dose reductions. QoL was not measured with a validated instrument; the improvement in symptoms and performance status is consistent with the preservation of QoL reported in HER2CLIMB [9,12]. The clinical course of both patients is summarized in Table 1.

**Table 1:** Treatment timeline, imaging findings, and clinical course of both patients IDC: Invasive ductal carcinoma; TCH: Docetaxel, carboplatin, and trastuzumab; RT: Radiotherapy; WBRT: Whole-brain radiotherapy; LVI: Lymphovascular invasion; PNI: Perineural invasion; EQD2: Equivalent dose in 2 Gy fractions; VAS: Visual Analog Scale; ECOG: Eastern Cooperative Oncology Group.

Date	Event/treatment	Findings/response
Case 1		
November 2022	Diagnosis: right breast IDC, cT4bN1M0; ER+, PR+, HER2 3+; BRCA-negative	PET-CT: no distant metastases
November 2022	Neoadjuvant TCH, one cycle (pertuzumab unaffordable)	>70% clinical reduction in lesion size
December 2022	Right modified radical mastectomy	ypT2N1; LVI+, PNI+; positive lateral margin
March 2023 to August 2023	Adjuvant TCH, 5 cycles; chest wall, axilla, and supraclavicular RT after cycle 2 (sandwich)	The patient completed treatment, and there was no evidence of local disease on clinical examination and no symptoms suggestive of metastatic disease.
September 2023 onward	Maintenance trastuzumab total eight cycles + tamoxifen	Patient progressed while on eighth course of maintenance inj trastuzumab and developed brain metastasis. Tab tamoxifen and inj trastuzumab were discontinued after progression.
January 2024	Neurological deficits during eighth maintenance trastuzumab cycle	MRI: bilateral cerebellar, left parietal and occipital metastases
February 2024 to March 2024	WBRT 50 Gy/25 fractions; ovarian ablation	Neurological symptoms improved after brain radiotherapy. Ovarian ablation was performed as she progressed on inj trastuzumab and in view of HR + disease. Ovarian ablation was performed by pelvic radiotherapy at a dose of 12 Gy in 4 fractions. The patient was given the option of medical or surgical oophorectomy, but she chose radiotherapy in view of financial unaffordability at that time.
March 2024 to September 2024	Lapatinib + capecitabine	Symptom control for approximately six months
September 2024	Seizures, neurological worsening; T-DM1 3.5 mg/kg started	MRI: right cerebellar and left parietal lesions, largest 1.9 × 3.5 × 1.5 cm
September 2024 to December 2024	Inj TDM1 started	Slight improvement initially
December 2024 to June 2025	Clinical progression on T-DM1; lapatinib + capecitabine rechallenge	Stabilization for approximately 6 months
June 2025	Right-sided limb weakness, slurred speech; conservative management	Required hospital admission for conservative management, and general condition improved.
July 2025	Tucatinib 300 mg twice daily + trastuzumab + capecitabine	Clinical improvement after two cycles; stable for seven months
February 2026	Neurological worsening; tucatinib stopped; three-weekly trastuzumab + pertuzumab started	MRI: stable lesion size, significant perilesional edema
April 2026	Last follow-up	Marked symptomatic improvement
Case 2		
October 2024	Diagnosis: right breast IDC, cT4bN3cM1 (lung, mediastinal nodes); ER/PR−, HER2 3+, Ki-67 55%; hemostatic single-fraction RT	6 × 6 cm ulcerated, bleeding mass
October 2024 to February 2025	TCH, six cycles	>70% clinical reduction of primary; PET-CT: lung progression
March 2025	Palliative RT 30 Gy/10 fractions + mold brachytherapy 21 Gy/7 fractions (EQD2 ~61 Gy); maintenance trastuzumab	Complete resolution of the primary breast lesion.
June 2025	Headache (VAS 4/10)	MRI: 6 mm right occipital peripherally enhancing lesion, suspected neurocysticercosis
September 2025	Worsening headache (VAS 6/10), vomiting after eight maintenance cycles	MRI: right parieto-occipital (5.1 × 4.5 × 5.4 cm) and right temporal (7.9 × 6.3 × 5.6 cm) lesions
September 2025 to November 2025	WBRT 56 Gy/28 fractions	Neurological symptoms improved.
November 2025	Tucatinib + trastuzumab + capecitabine	Resolution of headache and vomiting within the first two cycles
April 2026	Last follow-up, on tucatinib	Asymptomatic; ECOG 1 (baseline 2)

## Discussion

The patients in this case report were younger than 50 years and presented with biologically aggressive HER2-positive breast cancer with lymphovascular and perineural invasion in Case 1 and a high Ki-67 index with hormone receptor-negative disease in Case 2, features associated with early metastatic dissemination and CNS tropism [2,3]. Both had progressed on anti-HER2 agents with limited intracranial activity (trastuzumab, lapatinib, and T-DM1 in Case 1 and trastuzumab in Case 2) [4,5,14] before tucatinib-based therapy became accessible. This sequence reflects a common real-world challenge in resource-limited settings, where staggered drug availability and affordability frequently delay access to agents with proven CNS efficacy [16].

Despite later-line use, both patients showed improvement in neurological symptoms after initiation of tucatinib, trastuzumab, and capecitabine, with improvement in performance status and clinical stability over seven and five months of follow-up on tucatinib, respectively. These observations are consistent with the pivotal HER2CLIMB trial, which demonstrated significant PFS and OS benefit with tucatinib-based therapy across CNS subgroups, with preserved global QoL even in heavily pretreated, multiply progressed patients [8-12]. These findings are also supported by emerging real-world data. A large administrative claims analysis of tucatinib-based therapy, in which roughly 70% of patients had BMs, reported clinically meaningful treatment persistence across multiple prior lines of therapy, including after trastuzumab deruxtecan [17]. Similarly, a single-institution experience from a resource-limited setting demonstrated durable clinical benefit with HER2-targeted therapy in HER2-positive breast cancer with BMs despite restricted access to newer agents [18]. However, the improvement cannot be attributed to tucatinib alone: Case 2 began tucatinib shortly after whole-brain radiotherapy, which may have contributed to the response, and both patients had received prior brain radiotherapy and other systemic agents.

The response to tucatinib after lapatinib failure in Case 1 merits comment. Tucatinib is highly selective for HER2, with minimal inhibition of the epidermal growth factor receptor (EGFR), allowing effective HER2 inhibition with less EGFR-related toxicity [19], whereas lapatinib reaches BMs at highly variable concentrations, with tumor-to-serum ratios of 0.19 to 9.8 in resected breast cancer BMs [20]. Prior TKI exposure appears to attenuate, but not abolish, intracranial benefit: In TBCRC 022, the CNS objective response rate with neratinib plus capecitabine was 49% in lapatinib-naive and 33% in lapatinib-treated patients [21]. As HER2CLIMB excluded patients treated with lapatinib within the preceding 12 months [8], the improvement in Case 1 suggests that cross-resistance between HER2-targeted TKIs may be incomplete, although a single case cannot establish this.

Trastuzumab deruxtecan (T-DXd) has also shown substantial intracranial activity; in the DESTINY-Breast12 study, the intracranial objective response rate was 71.7%, and the 12-month PFS was 61.6% among patients with baseline BMs [22]. Tucatinib-based therapy remains active after T-DXd, with a median PFS of 4.7 months and OS of 13.4 months in a multicenter cohort of 101 patients, 38.6% of whom had BMs [23], and American Society of Clinical Oncology (ASCO) guidance supports tucatinib, trastuzumab, and capecitabine for patients with HER2-positive BMs, including those progressing after local therapy [24]. For Case 1, T-DXd was unaffordable at the time of intracranial progression, and the optimal sequence of these agents in patients with BMs remains uncertain even in high-income settings.

Neither patient received pertuzumab before CNS involvement, owing to cost. In the CLEOPATRA study, first-line pertuzumab delayed the onset of CNS metastases (median 15.0 versus 11.9 months) [25], and its omission may have contributed to the early CNS involvement observed here. Dual HER2 blockade also has modest activity in established BMs, with a CNS objective response rate of 11% and a clinical benefit rate of 68% at four months in the PATRICIA study [26], which supports the transition to trastuzumab and pertuzumab in Case 1. These gaps reflect broader access constraints in India, where only 57% of patients with HER2-positive breast cancer at a large public tertiary center received trastuzumab in 2016, despite marked improvement following the introduction of biosimilars [27].

Although neither patient in this case report has yet achieved a confirmed radiological complete response, deep and durable intracranial responses, including complete remission of clinical symptoms permitting transition to maintenance therapy, have been reported with tucatinib-based regimens, as supported by prospective trial data [9,11]. With longer follow-up, similarly favorable trajectories may be achievable in young, biologically responsive patients such as these.

This case report is limited by its small size, retrospective design, the absence of a validated QoL instrument, the lack of histological confirmation of brain lesions, the potential contribution of radiotherapy and other systemic agents, and the lack of formal Response Evaluation Criteria in Solid Tumors (RECIST) or Response Assessment in Neuro-Oncology Brain Metastases (RANO-BM) measurements [28], which were not applied, precluding definitive efficacy conclusions. Nonetheless, these cases support a role for tucatinib-based therapy, even when access constraints necessitate later-line use, and support efforts to expand earlier access to this regimen in similar settings.

## Conclusions

This real-world case report illustrates clinical and radiological improvement observed during tucatinib-based combination therapy (Tukavo™) in young patients with biologically aggressive, pretreated HER2-positive breast cancer and BMs, even when used in later treatment lines. The relative contribution of each treatment and the durability of response cannot be established from these two cases. These observations are consistent with pivotal trial findings in a real-world, resource-limited setting and support expanding timely access to tucatinib for patients with HER2-positive BMs.
